# Are inequalities in cancer diagnosis through emergency presentation narrowing, widening or remaining unchanged? Longitudinal analysis of English population-based data 2006–2013

**DOI:** 10.1136/jech-2017-210371

**Published:** 2018-11-08

**Authors:** Annie Herbert, Gary A Abel, Sam Winters, Sean McPhail, Lucy Elliss-Brookes, Georgios Lyratzopoulos

**Affiliations:** 1 ECHO (Epidemiology of Cancer Healthcare and Outcomes) Research Group, Department of Behavioural Sciences and Health, Institute of Epidemiology and Health Care, University College London, London, UK; 2 University of Exeter Medical School (Primary Care), Exeter, UK; 3 National Cancer Registration and Analysis Service (NCRAS), Public Health England, London, UK; 4 THIS (The Health Improvement Studies) Institute, and Cambridge Centre for Health Services Research, University of Cambridge, Institute of Public Health, Cambridge, UK

**Keywords:** cancer, inequalities, ageing, deprivation

## Abstract

**Background:**

Diagnosis of cancer through emergency presentation is associated with poorer prognosis. While reductions in emergency presentations have been described, whether known sociodemographic inequalities are changing is uncertain.

**Methods:**

We analysed ‘Routes to Diagnosis’ data on patients aged ≥25 years diagnosed in England during 2006–2013 with any of 33 common or rarer cancers. Using binary logistic regression we determined time-trends in diagnosis through emergency presentation by age, deprivation and cancer site.

**Results:**

Overall adjusted proportions of emergency presentations decreased during the study period (2006: 23%, 2013: 20%). Substantial baseline (2006) inequalities in emergency presentation risk by age and deprivation remained largely unchanged. There was evidence (p<0.05) of reductions in the risk of emergency presentations for most (28/33) cancer sites, without apparent associations between the size of reduction and baseline risk (p=0.26). If there had been modest reductions in age inequalities (ie, patients in each age group acquiring the same percentage of emergency presentations as the adjacent group with lower risk), in the last study year we could have expected around 11 000 fewer diagnoses through emergency presentation (ie, a nationwide percentage of 16% rather than the observed 20%). For similarly modest reductions in deprivation inequalities, we could have expected around 3000 fewer (ie, 19%).

**Conclusion:**

The proportion of cancer diagnoses through emergency presentation is decreasing but age and deprivation inequalities prevail, indicating untapped opportunities for further improvements by reducing these inequalities. The observed reductions in proportions across nearly all cancer sites are likely to reflect both earlier help-seeking and improvements in diagnostic healthcare pathways, across both easier-to-suspect and harder-to-suspect cancers.

## Introduction

Evidence from several countries and healthcare systems indicates that notable proportions of patients with cancer are diagnosed in an emergency context, and such patients tend to have poor clinical outcomes.^1–5^ Reducing the proportion of ‘emergency presenters’, and known related sociodemographic inequalities, is therefore desirable. In England, the percentage of patients with incident cancer diagnosed through emergency presentation has been decreasing, but whether previously reported disparities in the risk of such presentations are widening or narrowing has not been formally examined.^6^

Several hypotheses to explain the observed downward trends in emergency presentations can be considered, bearing in mind their complex and multifactorial aetiology.^1 7^ Trends in the proportion of emergency presentations are likely to reflect changes in patient and healthcare system factors, for example, increasing public awareness of likely cancer symptoms leading to earlier help-seeking, or wider availability and use of diagnostic investigations by general practitioners (GPs).^2 8^ Because the frequency of emergency presentations is substantially higher in older patients and those living in more deprived areas,^8^ it is important to establish whether these sociodemographic inequalities changed over time.^7 9–11^ In principle, awareness campaigns and efforts to improve diagnostic care may have resulted in either widening or narrowing of these inequalities.

We therefore aimed to examine changes over time in sociodemographic inequalities and cancer site variation in the proportion of cancers diagnosed through emergency presentation, to acquire insights about their potential aetiology and to inform potential targeting of interventions.^1 7^

## Methods

### Data

We longitudinally analysed population-based ’Routes to Diagnosis' data, on all cancer cases diagnosed at one of 33 different cancer sites, among patients aged 25 years and older in England during 2006–2013. The dataset was developed by the Public Health England National Cancer Registration and Analysis Service.^2^ It combines information from different data sources, including cancer registration, Hospital Episode Statistics, Cancer Waiting Times and National Health Service cancer screening programme (breast, bowel, cervical) data. It assigns diagnostic routes to all incident cancer cases, including emergency presentation, defined as diagnosis of cancer following an emergency hospital admission (including via GP, accident and emergency or bed bureau).^2^

Information was also available on year of diagnosis, sex, age group (25–49, 50–59, 60–69, 70–79, 80+ years of age), deprivation (five categories based on quintiles of the Income Domain of the 2010 version of the Index of Multiple Deprivation (IMD) of the Lower Super Output Area of the patient’s residence),^12^ cancer site (melanoma, brain, breast, lung, oral, oropharyngeal, thyroid, laryngeal, oesophageal, stomach, liver, renal, pancreatic, bladder, colon, small intestinal, rectal, anal, sarcoma, mesothelioma, multiple myeloma, Hodgkin’s lymphoma, non-Hodgkin’s lymphoma (NHL), leukaemia [acute lymphoblastic (ALL), acute myeloid (AML), chronic lymphocytic, chronic myeloid), cervical, uterine, prostate, testicular and cancer of unknown primary; the relevant International Statistical Classification of Diseases and Related Health Problems 10th revision (ICD-10) code definitions are included in online supplementary table S1).

10.1136/jech-2017-210371.supp1 Supplementary file 1

### Analysis

We aimed to examine time trends in emergency presentation and related sociodemographic inequalities and cancer site variation. We treated emergency presentation (vs diagnosis through any other diagnostic route) as a binary outcome. We performed adjusted analyses, by fitting two binary logistic regression models. Sex, age group, deprivation group and cancer site were all treated as categorical variables. We treated year as a continuous variable in both models in the main analysis, because this provided a single estimate (adjusted odds ratio [OR]) to represent overall time-trends. Initial analysis demonstrated that a categorical treatment of year provided a statistically significant improvement in the fit of models (that are described below) compared with a linear treatment of year, although it had little practical implication when considering the differences in the estimated inequality trends. Given that the additional benefit from a categorical treatment of year was limited and at the expense of a substantial increase in complexity of model estimates, in our main results we present the results of the model treating year as linear, but present those obtained using a categorical treatment of year as a sensitivity analysis (see the ’Results' section).

We first estimated adjusted associations (ORs) between year of diagnosis, sex, age group, deprivation group and cancer site and our outcome of interest (emergency presentation) by including these variables as main effects in a logistic regression model (with respective reference groups being 2006, male, 70–79 years, patients living in the least deprived areas and colon cancer; we treated year as a continuous variable [0, 1, …7] given an approximate linear relationship between year and proportions of emergency presentations). Using this model, we then predicted case-mix adjusted proportions of emergency presentations for each sex, age group, deprivation group and cancer site, in 2006 and 2013, i.e. the first and last study years, respectively.

We fitted a second model that had a similar structure to the first, and included the interaction terms sex*year, age group*year, deprivation*year, cancer site*year. This second model was used to formally examine whether inequalities by age and deprivation were changing over time, by inspecting the p values for the interaction terms age*year and deprivation*year, respectively. We then used this model to determine age group-specific and deprivation group-specific adjusted estimates in proportions of emergency presentations, by year. We also assessed the potential impact of hypothetical modest reductions in age and deprivation inequalities, by estimating the number of emergency presentations in 2013 (last year of the study period) had all patients had the same risk of emergency presentation as the adjacent age group with a lower risk of emergency presentation, and compared these with those observed in 2013. We then repeated the same analyses where all patients had the same risk of emergency presentation as the adjacent deprivation group with a lower risk of emergency presentation.

We additionally compared time-trends in proportions of emergency presentations by cancer site. The interaction term cancer site*year allowed us to formally test for changing inequalities between cancer sites, and estimate adjusted cancer site-specific proportions of emergency presentations per year. Finally, we determined whether baseline (2006) proportions of emergency presentation for each cancer site were associated with the degree of change in that outcome between 2006 and 2013 (ie, whether a higher baseline proportion was associated with a steeper decreasing trend, or vice versa), by estimating the Spearman’s rank correlation coefficient between the cancer-specific adjusted ORs of emergency presentation for 2013 vs 2006, and the cancer-specific odds of emergency presentation in 2006.

All analyses were carried out in Stata SE V.13.

## Results

During 2006–2013, there were 2 042 192 cases diagnosed with one of the 33 studied cancer sites, and among them 441 645 (21.6%) were diagnosed through emergency presentation.

The crude overall percentage of emergency presentations (across all cancer sites) decreased from 23.8% in 2006 to 20.0% in 2013 (table 1), with a crude OR for year of 0.97 (95% CI: 0.97 to 0.97) (see online supplementary table S2). The decrease was also evident after adjustment for case-mix variables as main effects (adjusted OR of 0.97, 95% CI: 0.97 to 0.97).

**Table 1:** Crude and adjusted proportions of emergency presentations (EPs)*, by sex, age group, deprivation quintile and cancer diagnosis, for patients diagnosed in 2006 and 2013

	2006				2013			
	No. of EPs	No. of cancer cases	*Crude %*	*Adjusted %*	No. of EPs	No. of cancer cases	*Crude %*	*Adjusted %*
**All cases**	56 551	237 415	*23.8*	*23.0*	55 313	276 569	*20.0*	*20.4*
Sex								
Female	26 418	117 696	*22.4*	*23.4*	25 907	135 095	*19.2*	*20.8*
Male	30 133	119 719	*25.2*	*22.7*	29 406	141 474	*20.8*	*20.0*
Age group (years)								
25–49	3199	23 733	*13.5*	*16.6*	3345	27 644	*12.1*	*16.0*
50–59	5386	34 264	*15.7*	*17.1*	4961	37 537	*13.2*	*15.5*
60–69	10 479	57 421	*18.2*	*18.2*	10 414	72 393	*14.4*	*15.4*
70–79	16 995	67 896	*25.0*	*22.6*	15 285	76 869	*19.9*	*18.9*
80+	20 492	54 101	*37.9*	*33.3*	21 308	62 126	*34.3*	*30.7*
Deprivation								
1 (least)	8744	46 474	*18.8*	*19.9*	9253	56 976	*16.2*	*17.9*
2	10 805	50 313	*21.5*	*21.5*	10 836	60 509	*17.9*	*18.9*
3	11 863	50 385	*23.5*	*22.6*	11 605	58 390	*19.9*	*19.9*
4	12 361	47 230	*26.2*	*24.3*	11 871	53 392	*22.2*	*21.7*
5 (most)	12 778	43 013	*29.7*	*26.9*	11 748	47 302	*24.8*	*23.5*
Cancer site								
Melanoma	241	8699	*2.8*	*3.3*	237	12 120	*2.0*	*2.3*
Breast	1859	38 913	*4.8*	*5.2*	1739	44 738	*3.9*	*4.4*
Oral	135	1924	*7.0*	*7.1*	134	2624	*5.1*	*5.8*
Thyroid	137	1617	*8.5*	*9.4*	182	2640	*6.9*	*7.7*
Uterine	536	6040	*8.9*	*9.4*	538	7420	*7.3*	*8.0*
Ororpharyngeal	123	1274	*9.7*	*10.4*	131	2261	*5.8*	*7.2*
Testicular	156	1524	*10.2*	*14.3*	143	1709	*8.4*	*12.0*
Prostate	3345	31 803	*10.5*	*10.4*	3134	40 146	*7.8*	*8.2*
Cervical	273	2335	*11.7*	*14.1*	260	2572	*10.1*	*11.4*
Laryngeal	215	1763	*12.2*	*11.6*	186	1807	*10.3*	*10.7*
Anal	99	765	*12.9*	*13.6*	131	1012	*12.9*	*13.2*
Sarcoma	171	1077	*15.9*	*16.5*	168	1522	*11.0*	*12.6*
HL	168	1056	*15.9*	*20.0*	212	1285	*16.5*	*19.0*
Rectal	1815	11 217	*16.2*	*15.5*	1441	11 182	*12.9*	*12.4*
Bladder	1723	8491	*20.3*	*17.0*	1536	8716	*17.6*	*15.4*
Oesophageal	1489	6451	*23.1*	*21.1*	1433	7215	*19.9*	*19.1*
CLL	632	2482	*25.5*	*23.8*	481	2908	*16.5*	*17.1*
NHL	2274	8405	*27.1*	*27.2*	2843	11 118	*25.6*	*25.5*
Renal	1594	5774	*27.6*	*27.4*	1775	8338	*21.3*	*23.0*
Ovarian	1806	5787	*31.2*	*32.9*	1591	6069	*26.2*	*27.6*
Colon	6524	19 431	*33.6*	*29.8*	6625	22 328	*29.7*	*26.7*
Stomach	2134	6346	*33.6*	*29.9*	1849	5603	*33.0*	*28.7*
CML	189	536	*35.3*	*35.2*	167	597	*28.0*	*29.3*
Mesothelioma	745	2086	*35.7*	*35.4*	736	2247	*32.8*	*30.5*
Multiple myeloma	1307	3563	*36.7*	*35.3*	1473	4642	*31.7*	*31.2*
Lung	12 735	32 680	*39.0*	*36.6*	12 653	36 247	*34.9*	*33.0*
Liver	1334	2774	*48.1*	*46.6*	1845	4300	*42.9*	*41.3*
Small intestine	360	746	*48.3*	*48.6*	533	1185	*45.0*	*44.7*
Pancreatic	3387	6718	*50.4*	*47.6*	3482	7804	*44.6*	*42.9*
AML	1115	2091	*53.3*	*50.8*	1246	2389	*52.2*	*51.1*
ALL	121	215	*56.3*	*59.6*	146	253	*57.7*	*61.5*
CUP	5618	9453	*59.4*	*54.0*	4172	7646	*54.6*	*49.6*
Brain	2191	3379	*64.8*	*69.2*	2091	3926	*53.3*	*59.2*

*Adjusted proportions estimated from a multivariable logistic regression model where outcome is EP (vs non-EP), and independent variables are sex, age group, deprivation, cancer site, year, age group*year, deprivation*year and cancer site*year (year entered as continuous variable both for main and interaction terms). The adjusted proportion in a given year was the predicted proportion of EPs, had the distribution of case-mix variables in that particular year been the same as that observed across all study years (2006–2013).

ALL, acute lymphoblastic leukaemia; AML, acute myeloid leukaemia; CLL, chronic lymphocytic leukaemia; CML, chronic myeloid leukaemia; CUP, cancer of unknown primary; HL, Hodgkin’s lymphoma; NHL, non-Hodgkin’s lymphoma.

After examining interaction terms between sociodemographic and cancer site variables and year of diagnosis, we found evidence of differential time-trends by age group (p<0.0001), deprivation (p=0.01) and cancer site (p<0.0001) but not by sex (p=0.66).

### Adjusted time-trends by age and deprivation

In 2006, there were notable inequalities in risk of emergency presentation by age and deprivation, with adjusted proportions of 17% vs 33% for 50–59 and 80+ year-old patients; and 20% vs 27% for patients living in the least and most deprived areas, respectively. These inequalities prevailed throughout the study period until 2013 (15% vs 31% for 50–59 and 80+ year-olds; and 18% vs 23% for patients living in the least and most deprived areas, respectively) (table 1; online supplementary table S2).

**Figure 1:**
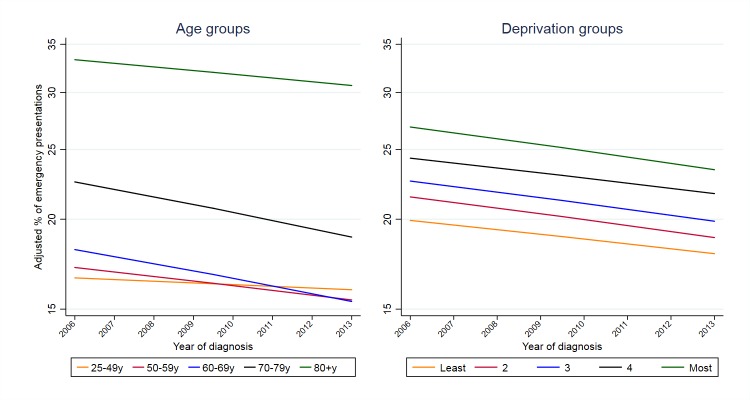
Time-trends in adjusted proportions of emergency presentations*, by age group and by deprivation group. *Adjusted proportions derived from logistic regression model where outcome is emergency presentation and independent variables are sex, age group, deprivation, cancer site, year and interaction terms for sex*year, age group*year, deprivation group*year and cancer site*year (year entered as a continuous variable both in main and interaction terms). The adjusted proportion in a given year was the predicted proportion of emergency presentations, had the distribution of case-mix variables in that particular year been the same as that observed across all study years (2006–2013). Trends are plotted on the log-proportions scale, to allow for a fair representation of *relative* changes over time between age and deprivation groups with different baseline frequencies of emergency presentation.

Adjusted proportions of emergency presentations decreased over time across all age groups, this decline being marginally faster for age groups 60–69 and 70–79 years (figure 1; table 2; online supplementary figure S1) with the range of age inequalities remaining fairly stable (figure 1; table 2). We estimated that had the risk of emergency presentation in each age group been that of the adjacent age group with a lower risk, there would have been 11 034 fewer emergency presentations in 2013 (table 2). That is, the overall proportion of emergency presentation in 2013 would have been 16% (44 279/276 569), instead of the observed 20%.

**Table 2:** Summary of age and deprivation inequalities as adjusted proportions and ORs of emergency presentations*, in 2006 and 2013; estimation of potentially avoidable (or ’excess') emergency presentations (last column, see footnote and the ’Methods' section)

	Adjusted %		Adjusted OR for 2013 vs 2006†	No. of emergency presentations in 2013	*No. of emergency presentations in 2013 that would be considered potentially avoidable (or ‘excess’), given modest reductions in inequalities*‡
	2006	2013			
Age group (years)					
25–49	16.6	16.0	0.93	3345	*150*
50–59	17.1	15.5	0.85	4961	*88*
60–69	18.2	15.4	0.78	10 414	*–*
70–79	22.6	18.9	0.76	15 285	*2861*
80+	33.3	30.7	0.85	21 308	*7935*
Total	–	–	*–*	55 313	11 034
Deprivation					
Least	19.9	17.9	0.76	9253	*–*
Second	21.5	18.9	0.73	10 836	*468*
Third	22.6	19.9	0.73	11 605	*663*
Fourth	24.3	21.7	0.74	11 871	*989*
Most	26.9	23.5	0.71	11 748	*962*
Total	–	–	*–*	55 313	*3081*

*Adjusted OR values in this column are derived as described in footnote '†'. Therefore, the presented adjusted OR values (2013 vs 2006) relate to the patient group defined by the reference category of each of the other main effect variables, ie, for each age group, they relate to patients who are male, living in least deprived areas, with colon cancer; and for deprivation group, they relate to patients who are male, aged 60–69 years, with colon cancer.

†Adjusted proportions and ORs estimated from a multivariable logistic regression model where outcome is emergency presentation (vs non- emergency presentation), and independent variables are sex, age group, deprivation, cancer site, year, age group*year, deprivation*year and cancer site*year (year entered as continuous variable both for main and interaction terms). The adjusted proportion in a given year was the predicted proportion of emergency presentations, had the distribution of case-mix variables in that particular year been the same as that observed across all study years (2006–2013).

‡Number of fewer cases of emergency presentations had each age and deprivation group had the same risk of emergency presentation as that of the adjacent group with a lower risk (eg, had those aged 50–59 years in 2006 had the same risk as those aged 25–49 years, ie, 16.5% rather than 17.0%). This was usually the younger age group or lower level deprivation group. However, in 2013, those aged 50–59 years had lower risks than those aged 25–49 years, and those aged 60–69 years had lower risks than those aged 50–59 years.

Adjusted proportions of emergency presentations generally decreased over time, similarly across all deprivation groups (table 2; figure 1; online supplementary figure S1), with the range of deprivation group inequalities remaining fairly stable between 2006 and 2013 (similar to what was observed for inequality by age group). We estimated that had the risk of emergency presentation in each deprivation group^2–5^ been that of the less deprived group adjacent to it, there would have been 3081 fewer emergency presentations in 2013 with the overall adjusted proportion of emergency presentations in 2013 being 19% (52 742/276 569) instead of 20%.

### Adjusted time-trends by cancer site

**Figure 2:**
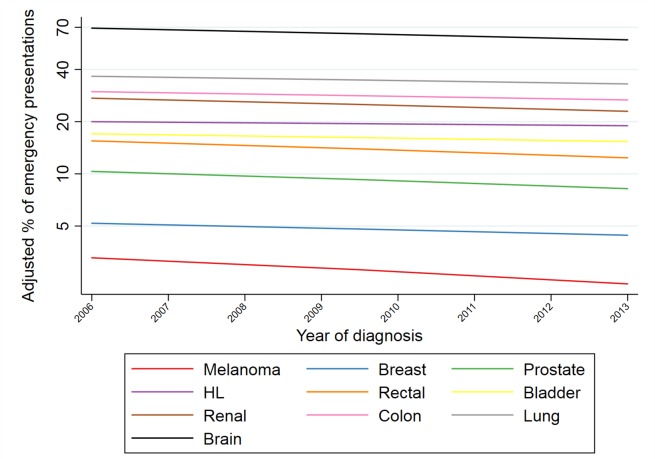
Time-trends in adjusted proportions of emergency presentations*, by cancer (shown for 10 different cancer sites). *Adjusted proportions derived from logistic regression model where outcome is emergency presentation and independent variables are sex, age group, deprivation, cancer site, year and interaction terms for sex*year, age group*year, deprivation group*year and cancer site*year (year entered as a continuous variable both in main and interaction terms). The adjusted proportion in a given year was the predicted proportion of emergency presentations, had the distribution of case-mix variables in that particular year been the same as that observed across all study years (2006–2013). Trends are plotted on the log-proportions scale, to allow a fair representation of *relative* changes over time between cancer sites with different baseline frequencies of emergency presentation. HL, Hodgkin’s lymphoma.

**Figure 3:**
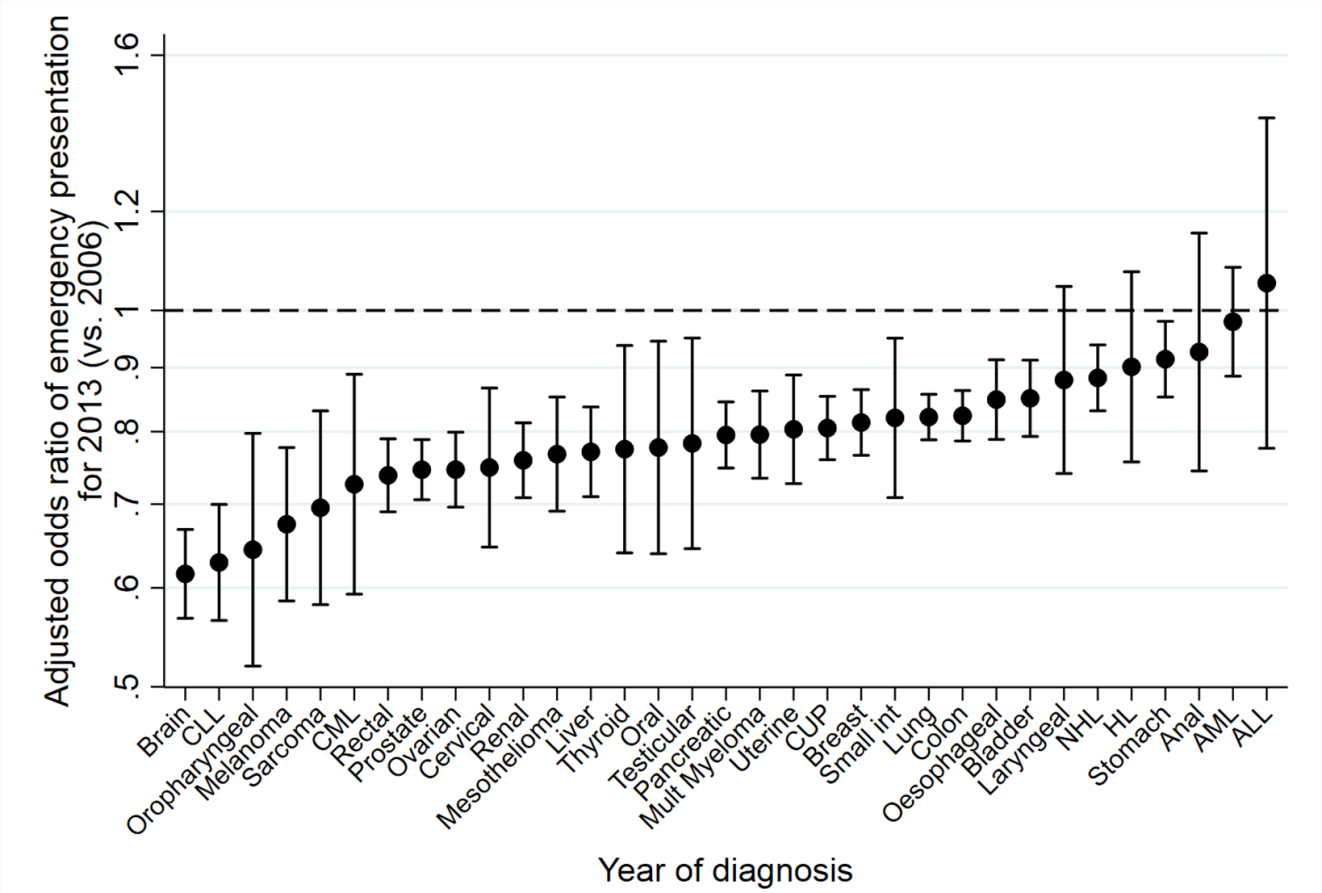
Cancer-specific adjusted ORs of emergency presentation* for 2013 (vs 2006). *Adjusted ORs derived from logistic regression model where outcome is emergency presentation and independent variables are sex, age group, deprivation, cancer site, year and interaction terms for sex*year, age group*year, deprivation group*year and cancer site*year (year entered as a continuous variable both in main and interaction terms). Therefore, the presented adjusted OR values (2013 vs 2006) relate to the patient group defined by the reference category of each of the other main effect variables, ie, for each cancer site (eg, brain), they relate to patients with that cancer who are male, aged 60–69 years and living in the least deprived areas. ORs are plotted on the log-odds scale, to allow a fair representation of *relative* differences between ORs. Bars represent 95% CIs. ALL, acute lymphoblastic leukaemia; AML, acute myeloid leukaemia; CLL, chronic lymphocytic leukaemia; CML, chronic myeloid leukaemia; CUP, cancer of unknown primary; HL, Hodgkin’s Lymphoma; NHL, non-Hodgkin lymphoma.

**Figure 4:**
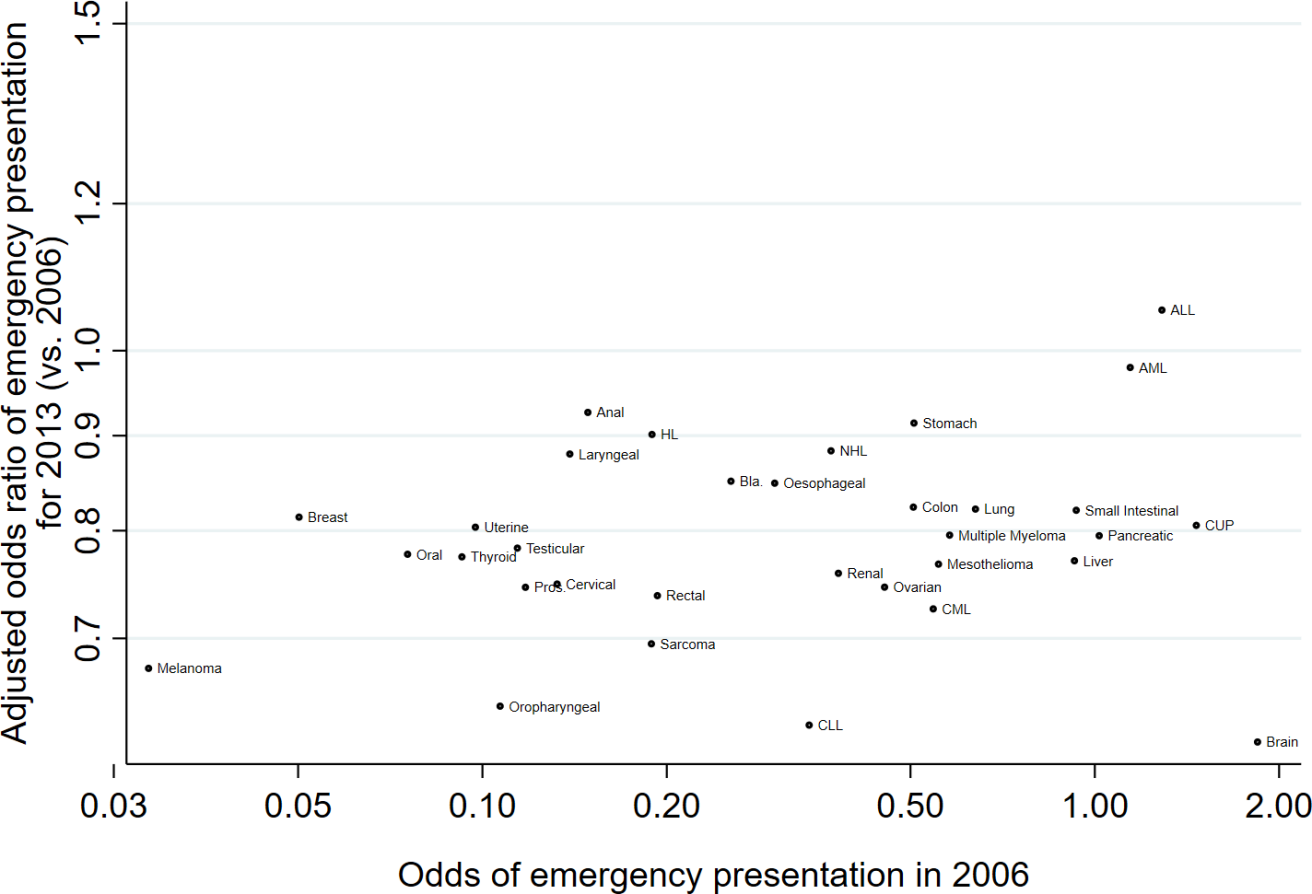
Scatter plot of adjusted ORs of emergency presentation* for 2013 (vs 2006) against odds of emergency presentation in 2006, by cancer site. *Adjusted ORs derived from logistic regression model where outcome is emergency presentation and independent variables are sex, age group, deprivation, cancer site, year and interaction terms for sex*year, age group*year, deprivation group*year and cancer site*year (year entered as a continuous variable both in main and interaction terms). Therefore, the presented adjusted OR values (2013 vs 2006) relate to the patient group defined by the reference category of each of the other main effect variables, ie, for each cancer site (eg, brain), they relate to patients with that cancer who are male, aged 60–69 years and living in the least deprived areas. ORs are plotted on the log-odds scale, to allow a fair representation of *relative* differences between ORs. ALL, acute  lymphoblastic leukaemia; AML, acute myeloid leukaemia; CLL, chronic lymphocytic leukaemia; CML, chronic myeloid leukaemia; CUP, cancer of unknown primary; HL, Hodgkin’s Lymphoma; NHL, Non-Hodgkin’s Lymphoma; Pros., Prostate.

Figure 2 illustrates trends in the adjusted proportions of emergency presentations with year of diagnosis, by cancer site, for 10 cancer sites (and also shown for all 33 sites in online supplementary figures S2 and S3). Considering each cancer site individually, there was evidence for decreasing time-trends for 28 of the 33 cancer sites studied (p<0.0001 to p=0.01 for these 28 sites), with adjusted ORs of emergency presentations for 2013 vs 2006 ranging from 0.62 (95% CI: 0.57 to 0.67) for brain to 0.91 (95% CI 0.85 to 0.98) for stomach cancer (figure 3). Among the five remaining sites without sufficient statistical evidence for decreasing trends, there was an apparent (but not statistically significant) increase in the proportions of emergency presentations over time for only one site: ALL (adjusted OR: 1.05, 95% CI: 0.78 to 1.43). Despite substantial variation in the time-trend ‘slope’ by cancer site, there was no evidence of an association between the change in risk of emergency presentation over time and baseline risk of emergency presentation for different cancer sites (figure 4; Spearman’s rank correlation coefficient between adjusted OR for emergency presentation for 2013 vs 2006 and odds of emergency presentation in 2006: *r*=0.20, 95% CI: −0.15 to 0.51; p=0.26).

### Sensitivity analysis

Repeating the main analysis by including diagnosis year as a categorical (as opposed to continuous) variable in the second model (which included both main effects and interaction terms), produced similar patterns of changes over time by age group, deprivation and cancer site, to those observed in main analysis (see online supplementary tables S3-S4 and figures S4-S5).

## Discussion

There were substantial reductions in the proportion of patients with cancer diagnosed with cancer through an emergency presentation but large baseline age and deprivation group inequalities in proportions of emergency presentations remained unchanged throughout the study period. Decreasing trends were observed across most cancer sites, without apparent associations with baseline cancer site-specific risk. In the latest study year, modest reductions in the age gradient in proportions of emergency presentations (ie, had the risk been reduced to that of the adjacent group with the lower risk) would have resulted in around 11 000 fewer cases of cancer being diagnosed as emergencies. Meanwhile, for similarly modest reductions in deprivation inequalities we could have expected around 3000 fewer.

### Strengths and limitations

We examined population-based data on over 2 million cancer cases from a recent 8-year period on patients with any of 33 different cancers, accounting for about 95% of all incident cancer cases. Beyond reductions in the percentage of emergency presentations, it should also be noted that the absolute number of cancer cases diagnosed through an emergency presentation also decreased. This minimises concerns about artefactually decreasing trends resulting from inflation of the number of incident cases due to potential overdiagnosis of certain cancer sites.^13 14^ It is likely that the observed decrease in emergency presentations partly reflects decreasing trends in the incidence of advanced stage disease,^3^ but we could not examine this hypothesis, given insufficiently complete data during most study years. Although increasing attendance rates at accident and emergency departments have been observed in recent periods,^15^ this factor is unlikely to have substantially biased the findings, as we have observed opposite (downward) trends in the proportion of patients with cancer diagnosed through emergency presentation.

Across the study years, patients were assigned to deprivation groups by classifying their small area of residence according to the Index of Multiple Deprivation scores of a single year (2010). Given that deprivation levels in a small area evolve over time (eg, due to redevelopment or inward/outward migration), this could have resulted in a degree of misclassification of the ‘true’ deprivation status of patients diagnosed in years other than 2010.^16–18^ We still, however, observe clear downward trends in emergency presentations across all (IMD 2010-defined) deprivation groups throughout the study period 2006–2013. Previous evidence on the impact of selective health migration on deprivation gradients in health outcomes such as mortality suggests that deprivation differences over time that may have been missed due to the above misclassification are likely to be small.^19^

### Comparison with other studies

A recent in-depth review documented that age and socioeconomic group inequalities exist in the risk of emergency presentation in a range of countries and health systems, but identified no evidence about time-trends in emergency presentations from countries other than England.^1 6 20^ Our study builds on these prior reports by formally examining adjusted trends in sociodemographic inequalities and cancer site variation.

### Implications for policy and practice and research

The study findings have several implications. Although biological/disease factors (such as tumour aggressiveness and malignant potential) influence the risk of emergency presentation, these are unlikely to change during a short period. Therefore, the observed decreases are likely to indicate changes in patient (e.g. help-seeking behaviour) or healthcare system factors, which appear to have been occurring across age and deprivation groups. However, partitioning the individual contribution of either patient or healthcare factors is challenging.

It should be acknowledged that age group inequalities in the risk of emergency presentation may, partly, reflect age-related variation in tumour biology. In contrast, deprivation group inequalities should theoretically be preventable in their near totality, as differences in disease factors by deprivation group are limited. Given that emergency presentation is associated with poorer clinical outcomes (such as survival) compared with other diagnostic routes,^2 3 21^ even modest reductions in age and deprivation group inequalities could lead to important population-wide public health gains. There is an increasing number of public health education campaigns about alarm symptoms that may indicate cancer.^9 22^ Whether these campaigns actually reduce inequalities is uncertain, and campaign effects on younger age groups and more affluent individuals are also likely.^22^ Several such interventions have taken place after the end of our study period, and future monitoring of trends in inequalities is needed.

Decreasing proportions of emergency presentations were observed for most cancer sites, without obvious associations with baseline (2006) risk, the availability of screening programmes for certain cancers (breast and colorectal in particular) or alarm symptom awareness campaigns in latter study years.^9^ Therefore, downward trends are likely to reflect changes in general attitudes about how quickly people seek medical help for new symptoms, or healthcare system improvements in diagnostic care (eg, greater use of specialist investigations or of urgent referral pathways for suspected cancer, thereby reducing the risk of emergency presentation during expectant management or while awaiting specialist assessment). These likely changes seem to have delivered benefits across the great majority of cancer sites.^23 24^ It is also possible that public health education campaigns about alarm symptoms, even if usually targeting specific cancer sites, have had broader benefits across cancer sites.

In conclusion, the proportion of patients with cancer diagnosed through an emergency presentation is decreasing across all age and deprivation groups and for most cancer sites, but inequalities between these patient groups prevail. It has been argued that earlier diagnosis initiatives should ’aim to reduce the proportion of patients with cancer who are diagnosed as emergencies to the absolute minimum dictated by tumour aggressiveness, having removed the potential influence of either healthcare or patient factors’.^7^ The findings therefore signal remaining opportunities to further decrease emergency presentations to the minimum levels dictated by tumour factors, through interventions targeting population subgroups at the greatest risk and continuing efforts to improve the quality of diagnostic care.

**What is already known on this subject**

- Many patients with cancer are diagnosed through an emergency presentation, which is associated with worse survival.
- There are large age and deprivation inequalities in risk of emergency presentation, with older patients and those living in more deprived areas being at higher risk.
- In England, reductions in the risk of emergency presentations have been described but whether baseline inequalities are widening or narrowing is not known.

**What this study adds**

- While there are welcome reductions in risk of emergency presentation, sociodemographic inequalities remained largely unchanged during 2006–2013.
- If there had been modest reductions in age and deprivation inequalities, respectively, in the final study year we could have expected 11 000 and 3000 fewer cancer diagnoses as emergencies, respectively, corresponding to overall nationwide percentages of emergency presentations of 16% and 19% instead of the observed 20%.

**Policy implications**

- The findings signal the potential for further reductions in the risk of emergency presentations by eliminating sociodemographic inequalities, particularly regarding deprivation gradients in this risk.
