# Supplementary material for: Are inequalities in cancer diagnosis through emergency presentation narrowing, widening or remaining unchanged? Longitudinal analysis of English population-based data 2006–2013
Source: J Epidemiol Community Health. 2018 Nov 8;73(1):3–10. doi: 10.1136/jech-2017-210371 (PMC6839789; doi:10.1136/jech-2017-210371)
Supplement: Supplementary file 1 [file jech-2017-210371supp001.pdf]

Table S1: ICD-10 codes used to define cancer sites in this study

Cancer site	ICD-10 code
Anal	C21.0-C21.2, C21.8
Acute lymphoblastic leukaemia (ALL)	C90.1
Acute myeloid leukaemia (AML)	C92.0, C92.4, C92.5, C93.0, C94.0, C94.2
Bladder	C67.0-C67.9
Brain	C71.0-C71.9
Breast	C50.0-C50.6, C50.8-C50.9
Cancer of Unknown primary (CUP)	C77.0-C77.5, C77.8-C78.8, C79.0-C79.8, C80
Cervical	C53.0, C53.1, C53.8, C53.9
Chronic lymphocytic leukaemia (CLL)	C91.1
Chronic myeloid leukaemia (CML)	C921
Colon	C18.0-C18.9
Hodgkin's Lymphoma (HL)	C81.0-C81.3, C81.7, C81.9
Laryngeal	C32.0-C32.3, C32.8, C32.9
Liver	C22.0-C22.4, C22.7, C22.9
Lung	C33, C34.0-C34.3, C34.8, C34.9
Melanoma	C43
Mesothelioma	C45.0-C45.2, C45.7, C45.9
Multiple Myeloma	C90.0-C90.2
Non-Hodgkin's Lymphoma (NHL)	C82.0-C82.2, C82.7, C82.9-C84.5, C85.0, C85.1, C85.7, C85.9
Oesophageal	C15.0-C15.5, C15.8, C15.9
Oral	C02.0-C02.4, C02.8-C03.1, C03.9-C04.1, C04.8, C04.9, C06.0-C06.2, C06.8, C06.9
Oropharyngeal	C01, C09.0, C09.1, C09.8-C10.4, C10.8, C10.9
Ovarian	C56, C57.0-C57.4, C57.7, C57.9
Pancreatic	C25.0-C25.4, C25.7-C25.9
Prostate	C61
Rectal	C19-C20
Renal	C64
Sarcoma*	C49.0-C49.6, C49.8, C49.9
Small intestine	C17.0-C17.3, C17.8, C17.9
Stomach	C16.0-C16.6, C16.8, C16.9
Testicular	C62.0, C62.1, C62.9
Thyroid	C73
Uterine	C54.0-C543, C54.8, C54.9, C55

*Both soft and connective tissue.

Table S2: Crude proportion, and crude and adjusted odds ratios (ORs) for diagnosis through emergency presentation*, by sex, age group, deprivation quintile, cancer diagnosis, in 2006-2013

				ORs			
	All cases	Number	(Crude %)	Crude	(95% CI)	Adjusted	(95% CI)
**All cases**	2042192	441645	(21.6)	.		.	
Sex							
Female	1005312	208275	(20.7)	(ref)			
Male	1036880	233370	(22.5)	1.1	(1.1, 1.1)	0.9	(0.9, 1.0)
Age							
25-49y	205239	25479	(12.4)	0.5	(0.5, 0.5)	0.7	(0.7, 0.7)
50-59y	278722	39420	(14.1)	0.6	(0.6, 0.6)	0.7	(0.7, 0.7)
60-69y	525372	83604	(15.9)	0.7	(0.7, 0.7)	0.7	(0.7, 0.7)
70-79y	572253	126735	(22.1)	(ref)			
80+y	460606	166407	(36.1)	2.0	(2.0, 2.0)	2.0	(2.0, 2.0)
Deprivation							
Least	410154	71435	(17.4)	(ref)			
2	443197	86487	(19.5)	1.1	(1.1, 1.1)	2.0	(2.0, 2.0)
3	430859	92035	(21.4)	1.3	(1.3, 1.3)	1.1	(1.1, 1.1)
4	400761	95824	(23.9)	1.5	(1.5, 1.5)	1.2	(1.2, 1.2)
Most	357221	95864	(26.8)	1.7	(1.7, 1.7)	1.3	(1.3, 1.4)
Cancer site**							
Melanoma	81936	1947	(2.4)	0.06	(0.06, 0.06)	0.07	(0.07, 0.07)
Breast	329884	14400	(4.4)	0.11	(0.11, 0.11)	0.12	(0.12, 0.12)
Oral	18211	1066	(5.9)	0.14	(0.13, 0.15)	0.19	(0.17, 0.20)
Thyroid	16512	1136	(6.9)	0.17	(0.16, 0.18)	0.23	(0.21, 0.24)
Uterine	53916	4323	(8.0)	0.20	(0.19, 0.21)	0.23	(0.22, 0.24)
Oropharyngeal	13496	899	(6.7)	0.17	(0.16, 0.18)	0.23	(0.21, 0.25)
Testicular	12665	1158	(9.1)	0.23	(0.22, 0.24)	0.37	(0.35, 0.39)
Prostate	280332	24615	(8.8)	0.22	(0.22, 0.22)	0.25	(0.24, 0.25)
Cervical	19474	2097	(10.8)	0.28	(0.27, 0.29)	0.36	(0.34, 0.37)
Laryngeal	14554	1512	(10.4)	0.27	(0.26, 0.29)	0.31	(0.29, 0.32)
Anal	7310	933	(12.8)	0.34	(0.32, 0.36)	0.38	(0.36, 0.41)
Sarcoma***	10080	1368	(13.6)	0.37	(0.35, 0.39)	0.42	(0.39, 0.44)
HL	9387	1513	(16.1)	0.45	(0.43, 0.48)	0.60	(0.57, 0.64)
Rectal	92024	12886	(14.0)	0.38	(0.37, 0.39)	0.40	(0.39, 0.41)
Bladder	69989	12763	(18.2)	0.52	(0.51, 0.53)	0.48	(0.47, 0.49)
Oesophageal	54923	11525	(21.0)	0.62	(0.61, 0.63)	0.63	(0.61, 0.64)
CLL	21540	4536	(21.1)	0.62	(0.60, 0.64)	0.63	(0.61, 0.66)
NHL	78623	20194	(25.7)	0.81	(0.79, 0.83)	0.91	(0.89, 0.93)
Renal	54545	13279	(24.3)	0.75	(0.73, 0.77)	0.85	(0.83, 0.86)
Ovarian	47162	13751	(29.2)	0.96	(0.94, 0.98)	1.10	(1.08, 1.13)
Colon	171142	51371	(30.0)	(ref)			
Stomach	48164	15561	(32.3)	1.11	(1.09, 1.13)	1.05	(1.03, 1.08)

CML	4365	1369	(31.4)	1.07	(1.00, 1.14)	1.22	(1.14, 1.30)
Mesothelioma	17564	5920	(33.7)	1.19	(1.15, 1.23)	1.26	(1.22, 1.30)
Multiple Myeloma	32355	11090	(34.3)	1.22	(1.19, 1.25)	1.28	(1.25, 1.31)
Lung	275164	101853	(37.0)	1.37	(1.35, 1.39)	1.37	(1.36, 1.39)
Liver	27601	12554	(45.5)	1.95	(1.90, 2.00)	2.05	(2.00, 2.11)
Small intestinal	7736	3559	(46.0)	1.99	(1.90, 2.08)	2.31	(2.21, 2.42)
Pancreatic	57510	27316	(47.5)	2.11	(2.07, 2.15)	2.17	(2.13, 2.21)
AML	17952	9344	(52.0)	2.53	(2.45, 2.61)	2.77	(2.68, 2.86)
ALL	1652	935	(56.6)	3.04	(2.75, 3.36)	4.16	(3.77, 4.60)
CUP	65339	37005	(56.6)	3.04	(2.98, 3.10)	2.85	(2.80, 2.91)
Brain	29085	17867	(61.4)	3.71	(3.61, 3.81)	4.88	(4.75, 5.01)
Year							
2006	237415	56551	(23.8)				
2007	240106	54507	(22.7)				
2008	247026	55122	(22.3)				
2009	252567	55168	(21.8)	0.97	(0.97, 0.97)	0.97†	(0.97, 0.97)
2010	255146	53515	(21.0)				
2011	261421	54804	(21.0)				
2012	271942	56665	(20.8)				
2013	276569	55313	(20.0)				

ALL = Acute Lymphoblastic Leukaemia; AML = Acute Myeloid Leukaemia; CI = Confidence Interval; CLL = Chronic Lymphocytic Leukaemia; CML = Chronic Myeloid Leukaemia; CUP = Cancer of Unknown Primary; HL = Hodgkin's Lymphoma; NHL = Non-Hodgkin's Lymphoma; OR = Odds Ratio; ref = reference category

*Adjusted odds ratios estimated from a multivariable logistic regression model where outcome is emergency presentation (vs. non-emergency presentation), and independent variables are sex, age group, deprivation, cancer site, and year (as a continuous variable).

**In ascending order of proportions of emergency presentations in 2006 – see Table 1.

***Both soft and connect tissue

†When fitting the same model but treating year of diagnosis as a categorical variable, the following adjusted OR estimates (all vs. 2006) for 2007, 2008, 2009, 2010, 2011, 2012, and 2013 were: 0.93, 0.91, 0.90, 0.86, 0.85, 0.84, and 0.80, respectively, without noticeable change in the OR values for all other variables. Based on this preliminary analysis, we treated year of diagnosis as a continuous variable for all subsequent analyses.

Figure S1. Adjusted odds ratio of emergency presentation* by age group (left) and deprivation group (right) by year.

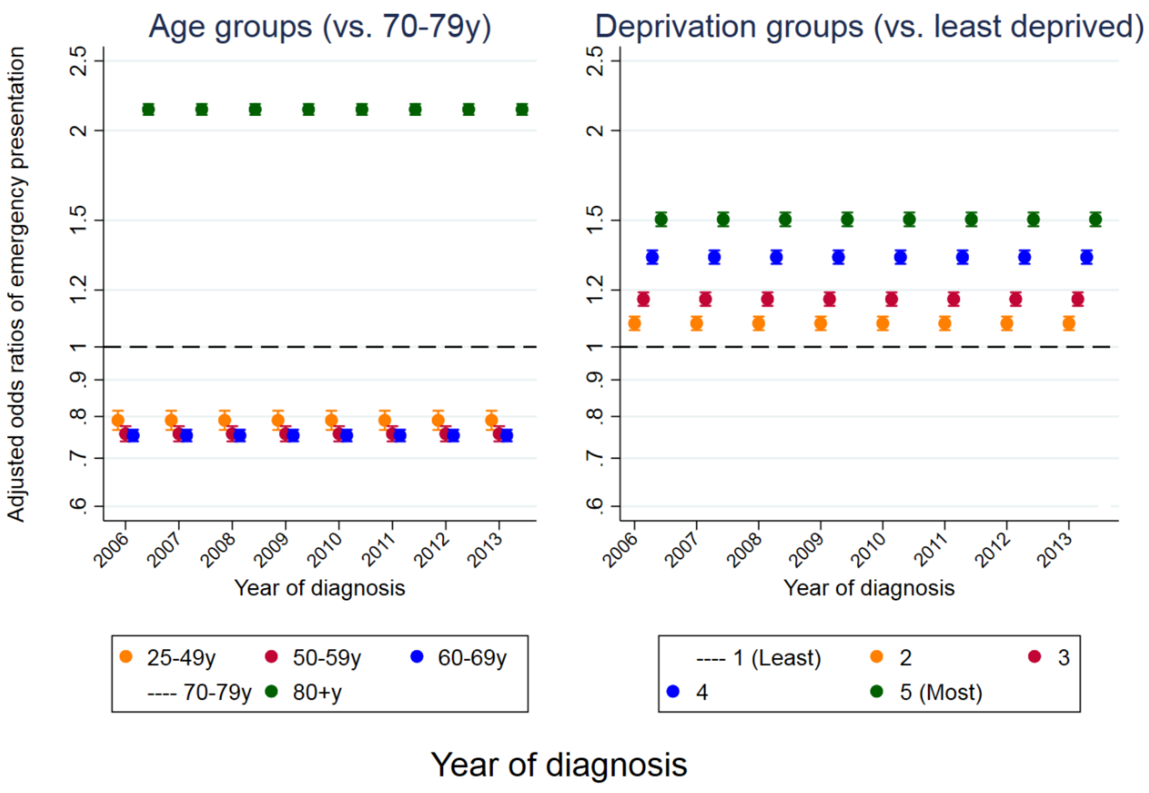

*Adjusted odds ratios derived from logistic regression model where outcome is emergency presentation and independent variables are sex, age group, deprivation, cancer site, year, and interaction terms for sex*year, age group*year, deprivation group*year and cancer site*year (year entered as a continuous variable both in main and interaction terms).

Figure S2. Trends in adjusted proportion of emergency presentation* by cancer site for percentages in 2006 that are less than 25 (y-axis on logarithmic scale).

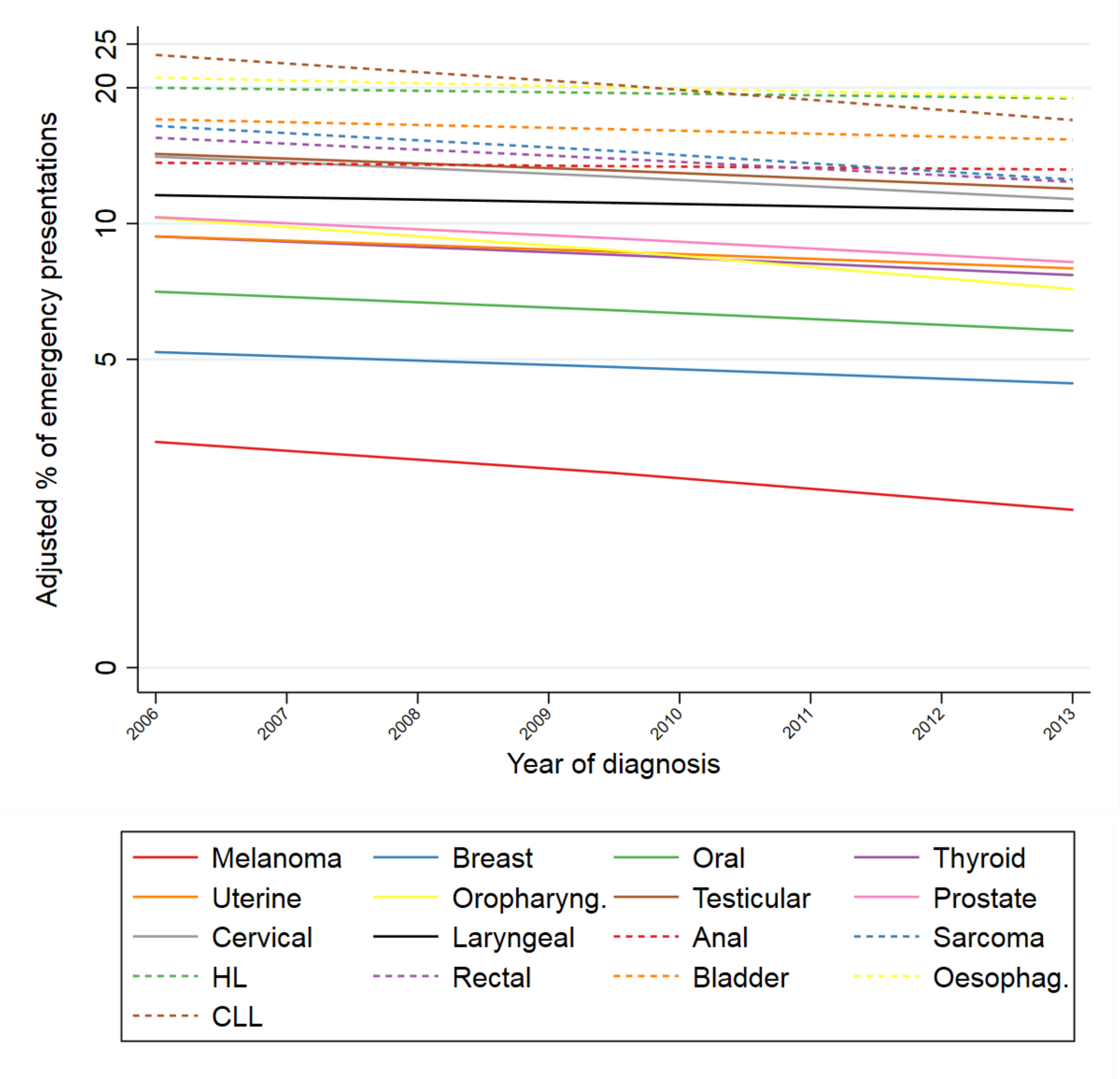

CLL = Chronic Lymphocytic Leukaemia; HL = Hodgkin's Lymphoma; Oesophag. = Oesophageal; Oropharyng. = Oropharyngeal

*Adjusted proportions derived from logistic regression model where outcome is emergency presentation and independent variables are sex, age group, deprivation, cancer site, year, and interaction terms for sex*year, age group*year, deprivation group*year and cancer site*year (year entered as a continuous variable both in main and interaction terms). These adjusted proportions were estimated given a case-mix in each year that was distributed as for the entire study period.

Figure S3. Trends in adjusted proportion of emergency presentation* by cancer site for percentages in 2006 that are 25 or greater (y-axis on logarithmic scale).

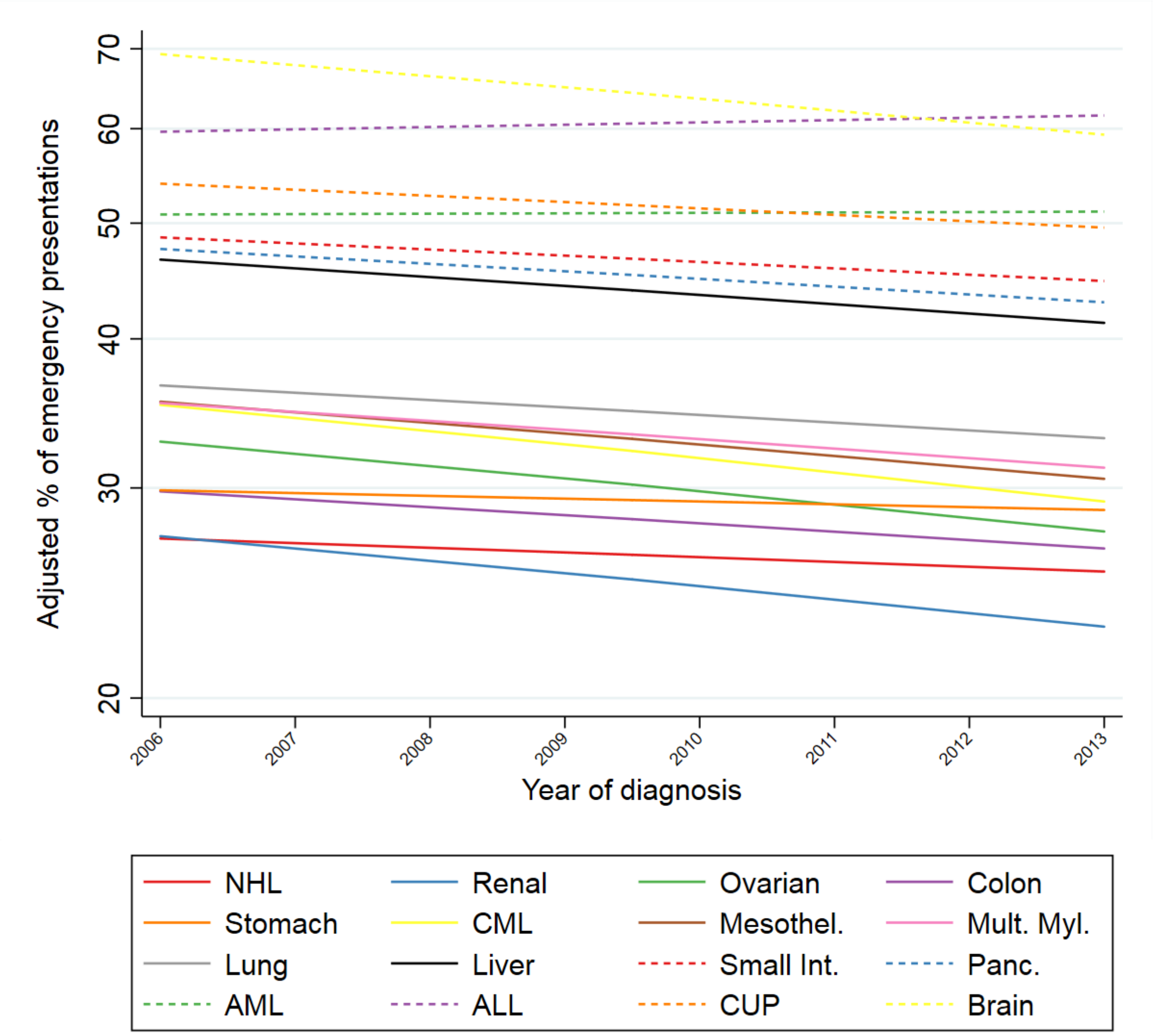

ALL = Acute Lymphoblastic Leukaemia; AML = Acute Myeloid Leukaemia; CML = Chronic Myeloid Leukaemia; CUP = Cancer of Unknown Primary; Mesothel. = Mesothelioma; Mult. Myl. = Multiple Myeloma; NHL = Non-Hodgkin's Lymphoma; Panc. = Pancreatic; Small Int. = Small Intestinal

*Adjusted proportions derived from logistic regression model where outcome is emergency presentation and independent variables are sex, age group, deprivation, cancer site, year, and interaction terms for sex*year, age group*year, deprivation group*year and cancer site*year (year entered as a continuous variable both in main and interaction terms). These adjusted proportions were estimated given a case-mix in each year that was distributed as for the entire study period.

Table S3: Adjusted proportions of emergency presentations (EPs)* for patients diagnosed in 2006 and 2013 - when model includes year as a continuous variable and as a categorical variable, respectively

	2006		2013	
	**Adjusted % when year continuous****	**Adjusted % when year categorical*****	**Adjusted % when year continuous****	**Adjusted % when year categorical*****
**All cases**	23.0	23.4	20.4	20.4
Sex				
Female	23.4	23.6	20.8	20.8
Male	22.7	23.2	20.0	20.0
Age-group (years)				
25-49	16.6	17.3	16.0	16.4
50-59	17.1	17.8	15.5	15.8
60-69	18.2	18.6	15.4	15.5
70-79	22.6	22.9	18.9	18.9
80+	33.3	33.5	30.7	30.4
Deprivation				
1 (Least)	19.9	20.2	17.9	17.8
2	21.5	21.8	18.9	18.8
3	22.6	23.1	19.9	20.0
4	24.3	24.7	21.7	21.7
5 (Most)	26.9	27.4	23.5	23.7
Cancer site				
Melanoma	3.3	3.3	2.3	2.2
Breast	5.2	5.3	4.4	4.2
Oral	7.1	7.5	5.8	5.5
Thyroid	9.4	10.3	7.7	8.2
Uterus	9.4	9.5	8.0	7.7
Oropharyngeal	10.4	11.9	7.2	7.4
Testicular	14.3	14.8	12.0	11.2
Prostate	10.4	10.6	8.2	8.5
Cervical	14.1	13.8	11.4	11.5
Laryngeal	11.6	12.9	10.7	11.0
Anal	13.6	13.4	13.2	13.5
Sarcoma	16.5	16.4	12.6	11.6
HL	20.0	19.3	19.0	19.0
Rectal	15.5	15.8	12.4	12.9
Bladder	17.0	18.1	15.4	15.8
Oesophageal	21.1	22.0	19.1	19.2
CLL	23.8	24.0	17.1	16.1
NHL	27.2	27.8	25.5	26.0
Renal	27.4	27.8	23.0	21.9
Ovarian	32.9	32.6	27.6	27.1
Colon	29.8	31.4	26.7	27.9
Stomach	29.9	30.5	28.7	29.9
CML	35.2	35.6	29.3	28.6
Mesothelioma	35.4	35.8	30.5	31.5
Multiple	35.3	35.6	31.2	30.8
Lung	36.6	36.7	33.0	32.8
Liver	46.6	46.1	41.3	41.0
Small intestine	48.6	49.1	44.7	45.3
Pancreatic	47.6	48.0	42.9	42.4
AML	50.8	52.7	51.1	51.1
ALL	59.6	60.5	61.5	60.7
CUP	54.0	54.8	49.6	49.9
Brain	69.2	67.7	59.2	56.0

ALL = Acute Lymphoblastic Leukaemia; AML = Acute Myeloid Leukaemia; CLL = Chronic Lymphocytic Leukaemia; CML = Chronic Myeloid Leukaemia; CUP = Cancer of Unknown Primary; HL = Hodgkin's Lymphoma; NHL = Non-Hodgkin's Lymphoma

*Adjusted proportions estimated from a multivariable logistic regression model where outcome is emergency presentation (vs. non-emergency presentation), and independent variables are sex, age group, deprivation, cancer site, year, age group*year, deprivation*year,

and cancer site*year. These adjusted proportions were estimated given a case-mix in each year that was distributed as for the entire study period.
**Year entered as continuous variable both for main and interaction terms.
***Year entered as categorical variable both for main and interaction terms.

Table S4: Summary of age and deprivation inequalities as adjusted proportions and odds ratios of emergency presentations *, in 2006 and 2013, and estimation of potentially avoidable (or 'excess') emergency presentations - when model includes year as a categorical variable

	Adjusted %				
	**2006**	**2013**	**Adjusted OR for 2013 vs. 2006**	**No. of emergency presentations in 2013**	***No. of emergency presentations in 2013 that would be considered potentially avoidable (or 'excess'), given modest reductions in inequalities****
**Age**					
25-49y	17.3	16.4	1.01	3345	*132*
50-59y	17.8	15.8	0.92	4961	*95*
60-69y	18.6	15.5	0.84	10414	-
70-79y	22.9	18.9	0.82	15285	*2800*
80+y	33.5	30.4	0.92	21308	*7905*
**Total**	**-**	**-**	**--**	**55313**	*10931*
**Deprivation**					
Least	20.2	17.8	0.82	9253	-
2nd	21.8	18.8	0.79	10836	*545*
3rd	23.1	20.0	0.80	11605	*748*
4th	24.7	21.7	0.81	11871	*915*
Most	27.4	23.7	0.78	11748	*966*
**Total**	**-**	**-**	**-**	**55313**	*3175*

OR = Odds ratio

*Adjusted proportions and odds ratios estimated from a multivariable logistic regression model where outcome is emergency presentation (vs. non- emergency presentation), and independent variables are sex, age group, deprivation, cancer site, year, sex*year, age group*year, deprivation*year, and cancer site*year (year entered as categorical variable both for main and interaction terms). Adjusted proportions were estimated given a case-mix in each year that was distributed as for the entire study period.

**Number of fewer cases of emergency presentation had each age and deprivation group had the same risk of emergency presentation as that of the adjacent group with a lower risk (e.g. had 50-59y olds in 2006 had the same risk as 25-49y olds, i.e.17.3% rather than 17.8%). This was usually the younger age group or lower level deprivation group. However, for 2013, 50-59y olds had lower risks than 25-49y olds, and 60-69y olds had lower risks than 50-59y olds, and so in this year for 25-59y olds we assigned the risk of the next oldest age-group.

Figure S4: Time-trends in adjusted proportions of emergency presentations*, by age group and by deprivation group - when model includes year as a categorical variable

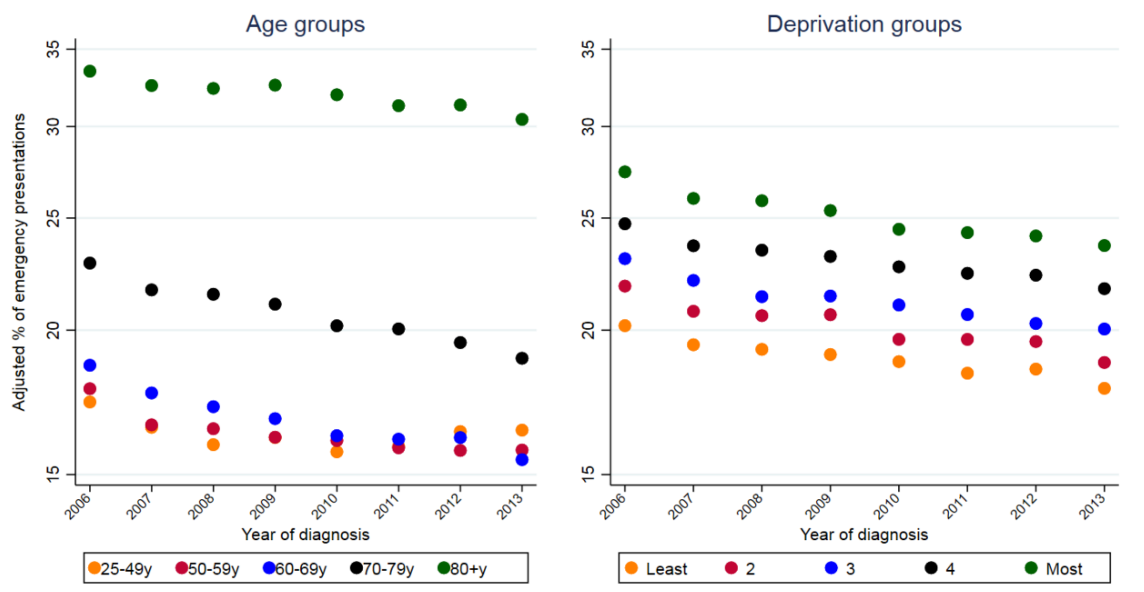

Trends are plotted on the log-proportions scale, to allow for a fair representation of *relative* changes over time between age and deprivation groups with different baseline frequency of emergency presentation.

*Adjusted proportions derived from logistic regression model where outcome is emergency presentation and independent variables are sex, age group, deprivation, cancer site, year, and interaction terms for sex*year, age group*year, deprivation group*year and cancer site*year (year entered as a categorical variable both in main and interaction terms). These adjusted proportions were estimated such that the case-mix within each study year was fixed to be the same as for the entire study period.

Figure S5: Time-trends in adjusted proportions of emergency presentations*, by cancer - when model includes year as a categorical variable

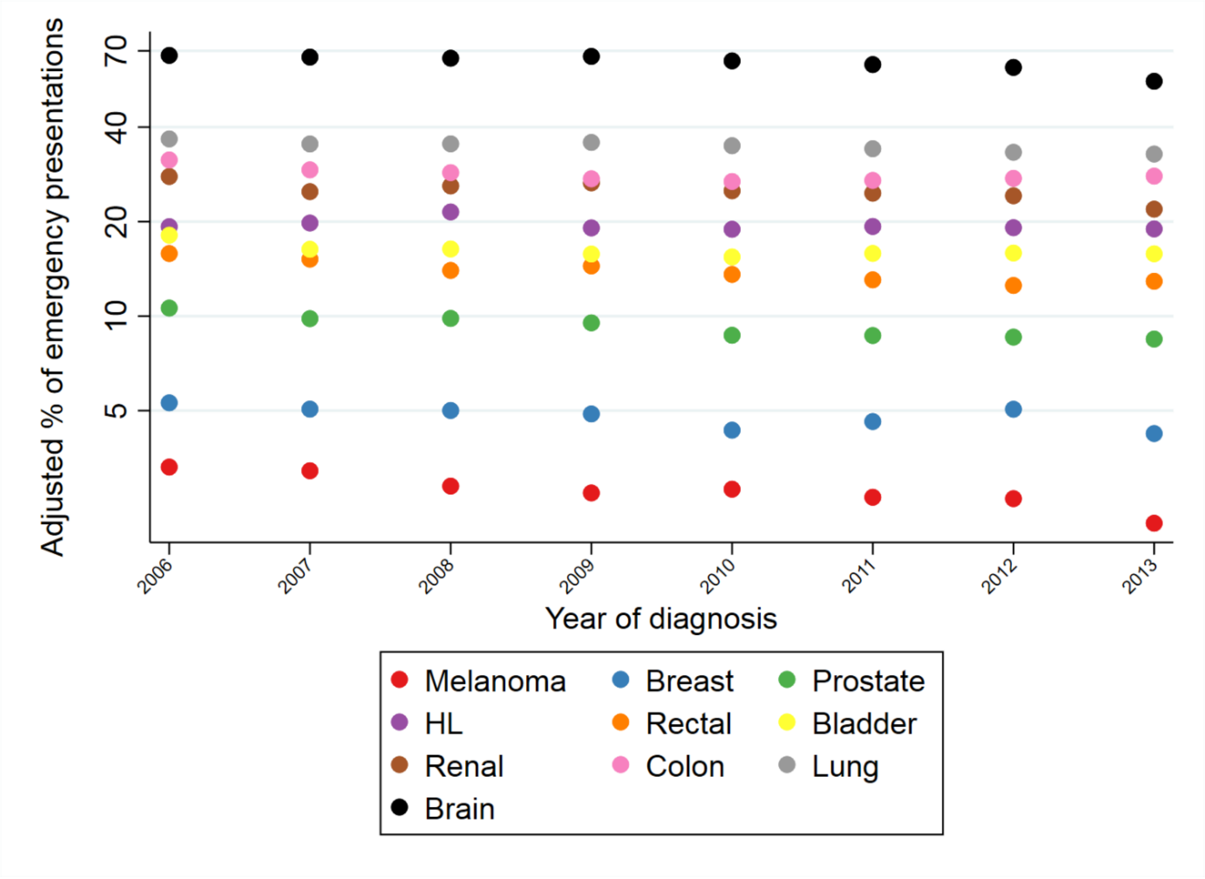

Trends are plotted on the log-proportions scale, to allow a fair representation of *relative* changes over time between cancer sites with different baseline frequency of emergency presentation.

H Lymph = Hodgkin's Lymphoma

*Adjusted proportions derived from logistic regression model where outcome is emergency presentation and independent variables are sex, age group, deprivation, cancer site, year, and interaction terms for sex*year, age group*year, deprivation group*year and cancer site*year (year entered as a continuous variable both in main and interaction terms). These adjusted proportions were estimated such that the case-mix within each study year was fixed to be the same as for the entire study period.
